# Automated Alignment of Multi-Modal Plant Images Using Integrative Phase Correlation Approach

**DOI:** 10.3389/fpls.2018.01519

**Published:** 2018-10-16

**Authors:** Michael Henke, Astrid Junker, Kerstin Neumann, Thomas Altmann, Evgeny Gladilin

**Affiliations:** Molecular Genetics, Leibniz Institute of Plant Genetics and Crop Plant Research (IPK), Gatersleben, Germany

**Keywords:** high-throughput plant phenotyping, automated image analysis, multi-modal image registration, affine transformations, non-uniform motion, Fourier-Mellin phase correlation

## Abstract

Modern facilities for high-throughput phenotyping provide plant scientists with a large amount of multi-modal image data. Combination of different image modalities is advantageous for image segmentation, quantitative trait derivation, and assessment of a more accurate and extended plant phenotype. However, visible light (VIS), fluorescence (FLU), and near-infrared (NIR) images taken with different cameras from different view points in different spatial resolutions exhibit not only relative geometrical transformations but also considerable structural differences that hamper a straightforward alignment and combined analysis of multi-modal image data. Conventional techniques of image registration are predominantly tailored to detection of relative geometrical transformations between two otherwise identical images, and become less accurate when applied to partially similar optical scenes. Here, we focus on a relatively new technical problem of FLU/VIS plant image registration. We present a framework for automated alignment of FLU/VIS plant images which is based on extension of the phase correlation (PC) approach − a frequency domain technique for image alignment, which relies on detection of a phase shift between two Fourier-space transforms. Primarily tailored to detection of affine image transformations between two structurally identical images, PC is known to be sensitive to structural image distortions. We investigate effects of image preprocessing and scaling on accuracy of image registration and suggest an integrative algorithmic scheme which allows to overcome shortcomings of conventional single-step PC by application to non-identical multi-modal images. Our experimental tests with FLU/VIS images of different plant species taken on different phenotyping facilities at different developmental stages, including difficult cases such as small plant shoots of non-specific shape and non-uniformly moving leaves, demonstrate improved performance of our extended PC approach within the scope of high-throughput plant phenotyping.

## 1. Introduction

In recent years, plant phenotyping became an indispensable analytical tool in quantitative plant sciences. Modern multi-camera systems such as LemnaTec-Scanalyzer3D (LemnaTec GmbH, Aachen, Germany) enable acquisition of large amount of multi-modal image data, including visible light (VIS), fluorescence (FLU), and near-infrared (NIR) images. To derive reliable quantitative traits of plant morphology, development and functions from large amount of multi-modal image data, efficient algorithmic solutions for detection and quantification of plant structures are required (Minervini et al., 2015).

**Figure 1:**
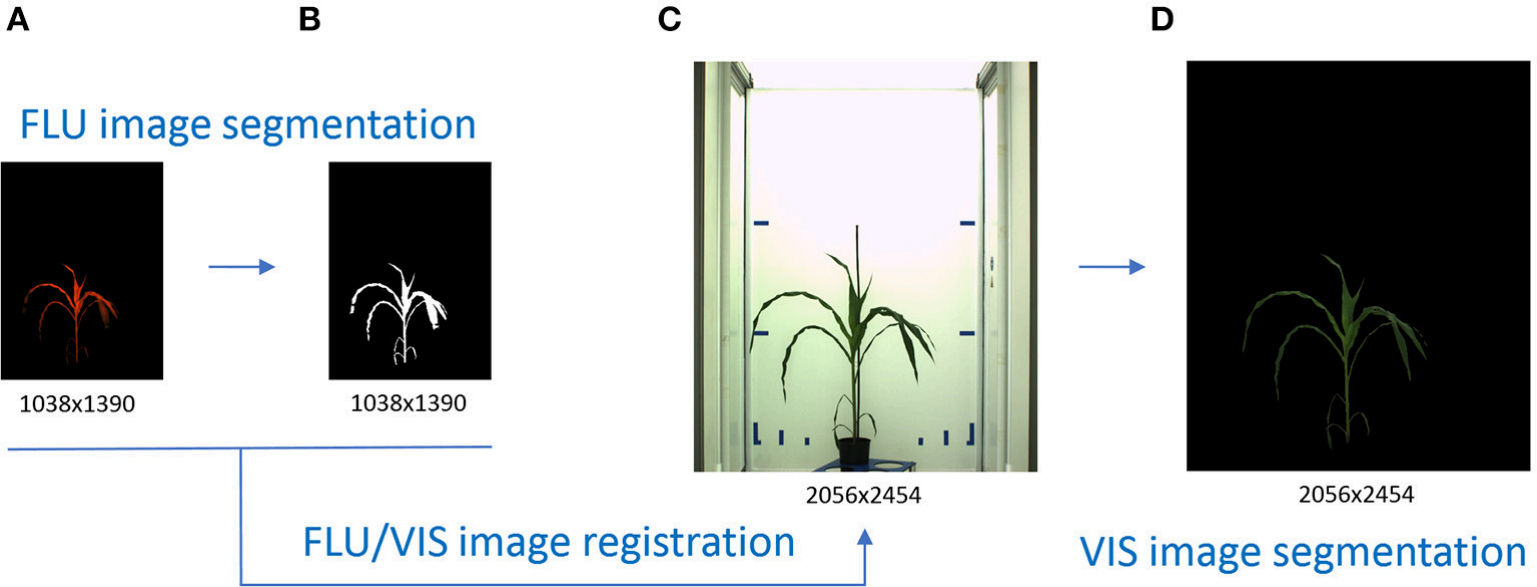
Principle scheme of VIS image segmentation by means of VIS/FLU image alignment. Higher plant-background contrast enables a straightforward segmentation of FLU images **(A)** resulting in a binary mask of the plant region **(B)**. Inhomogeneous illumination and visibility of diverse background structures challenge an accurate segmentation of VIS images **(C)**. By applying the binary mask of the registered FLU image, automated segmentation of the VIS image **(D)** is performed.

Quantitative analysis of plant images begins with image segmentation which aims to identify image regions corresponding to whole plant or particular plant organs. Reliability of phenotypic plant traits essentially depends on accuracy and robustness of image segmentation algorithms. Straightforward segmentation of VIS plant images by means of global thresholding is often hampered by a number of natural and technical reasons including variable plant coloring, inhomogeneous illumination, shadows and reflections in plant and background regions. Differently from VIS, intensity of FLU images strongly correlates with chlorophyll content of plant structures which provides a natural contrast to chlorophyll-free background regions. Higher contrast between intensity of plant and background regions makes fluorescent images to a natural reference for detection and segmentation of plant structures. Once appropriately aligned, the binary mask of segmented FLU images can be applied for segmentation of VIS images. Such a segmentation-via-registration scheme has a considerable advantage of being generic and avoids diverse difficulties by the segmentation of structurally more complex and variable VIS images, see Figure 1.

**Figure 2:**
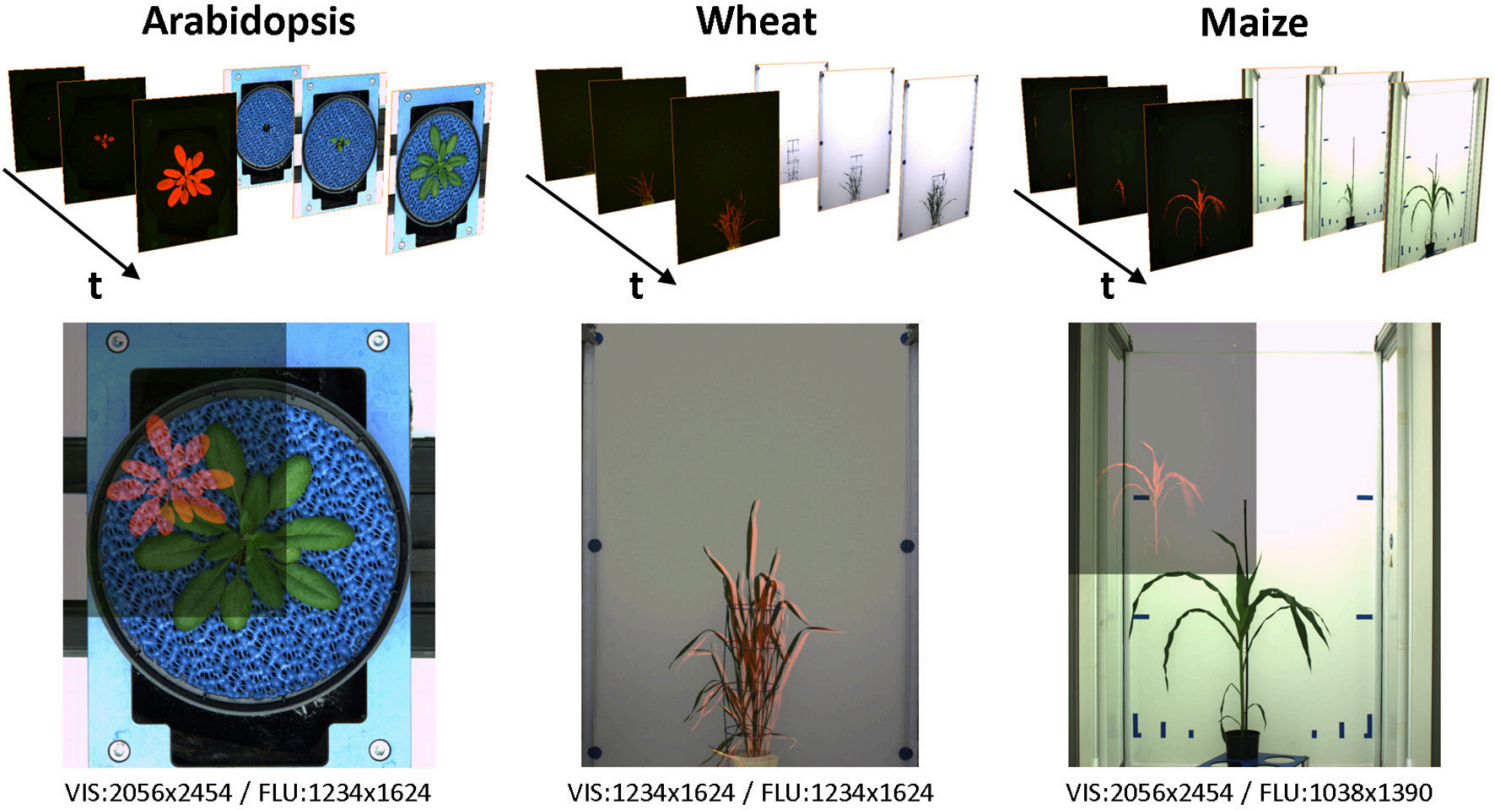
Examples of VIS/FLU images of different plant species acquired from high-throughput plant phenotyping experiments. Due to differences in camera resolution and position, multi-modal images exhibit differences in the relative size, position, and spatial orientation. Blends of images pairs in the lower row show differences of the VIS/FLU resolution.

Two images of different modalities may, in general, differ by a relative affine transformation (i.e., translation, rotation and scaling), but also structurally. For example, contours of walls, carriers and other light reflecting/absorbing objects in VIS images are typically not present in FLU images, see Figure 2. Consequently, alignment of multi-modal images is associated with the problem of finding correspondences between two structurally non-identical images that exhibit only partial similarities.

A broad spectrum of methods for image registration has been previously developed in context of biomedical and geographic imaging (Zitova and Flusser, 2003; Xiong and Zhang, 2010; Lahat et al., 2015; Brock et al., 2017; Goshtasby, 2017). To establish correspondences between two images, manually or automatically generated landmarks (spatial feature-points), intensity information or frequency-domain features were used. The frequency-space based techniques such as Fourier-Mellin phase correlation (PC) rely on the Fourier-shift theorem, which enables detection of a spatial shift in Cartesian or polar systems of coordinates from the phase-shift of their Fourier transforms (Kuglin and Hines, 1975; Reddy and Chatterji, 1996; Wolberg and Zokai, 2000). From previous works (Stone et al., 2001; Foroosh et al., 2002; Argyriou and Vlachos, 2006), it is known that PC is surprisingly robust with respect to noise, but becomes less accurate in presence of multiple structurally similar patterns or considerable structural distortions such as non-rigid image transformations (e.g., deformation, non-uniform motion, etc.). Requirements of additional pre-processing steps by applying PC for registration of non-identical and multi-modal images were reported in Wisetphanichkij and Dejhan (2005), Wang et al. (2013), Gladilin and Eils (2015), and Almonacid-Caballer et al. (2017).

Applications of image registration techniques in context of plant image analysis are still relatively scarce (De Vylder et al., 2012; Raza et al., 2015). Structural differences between multi-modal plant images and presence of non-uniform image motion due to uncorrelated movements of leaves make alignment of multi-modal plant images a challenging task. Here, we are concerned with investigation of diverse facets of multi-modal plant image alignment and suggest extensions to the conventional single-step PC approach for improved robustness and accuracy of FLU/VIS image registration.

## 2. Methods

### 2.1. Image acquisition

Time-series of VIS and FLU top-/side-view images of developing maize, wheat and arabidopsis shoots were acquired from high-throughput experiments performed over more than 2 weeks using LemnaTec-Scanalyzer3D high-throughput phenotypic platforms (LemnaTec GmbH, Aachen, Germany). In the highest expansion stage, the LemnaTec Scanalyzer3D consists of three measuring boxes, each equipped with one (or more) different sensor system. Following a measuring plan, plants are moved automatically from the greenhouse to the measuring facility where they are successively transported from one measuring box (e.g., VIS) to the next one (e.g., FLU). Corresponding VIS and FLU images are therefore taken within few seconds one after another, which are required to move the plants from the VIS to the FLU measuring box, respectively. Table 1 summarizes image data modalities and formats used in this study.

**Table 1:** An overview of image data used in this study including three different experiments of three different species, each taken in visible light and fluorescence, obtained by three different LemnaTec high-throughput phenotyping facilities for large, intermediate size, and small plants at the IPK Gatersleben.

** Species, views**	**# Plants**	**# Days**	**# Angles**	**# VIS/FLU pairs**	**VIS size**	**FLU size**
Arabidopsis, top	4	20	1	80	2,056 × 2,454	1,234 × 1,624
Wheat, side	4	47	3	564	1,234 × 1,624	1,234 × 1,624
Maize, side	6	22	4	526	2,056 × 2,454	1,038 × 1,390

### 2.2. Image preprocessing

To increase the robustness of PC calculation, FLU images are uniformly pre-scaled to the height of VIS images prior to affine PC registration. In order to assess effects of structural differences between VIS and FLU images on accuracy and robustness of PC registration, evaluation tests were carried out with original as well as manually segmented images. Manual segmentation was performed using variable cut-off thresholds for different background regions followed by a subsequent manual removal of remaining structural artifacts. Since PC is known to rely on edge information, edge images were generated using color-edge algorithm (Henriques, 2010) and used in addition to grayscale images for finding global affine transformations. Furthermore, image scaling and cropping was introduced to investigate effects of absolute and relative image size on accuracy and robustness of PC registration. In cropped images, the crop-mask was defined by the dimension of the bounding box of all manually segmented plant structures for a particular day of experiment, i.e., the developmental stage of the plant. No further preprocessing steps were applied with exception of Arabidopsis images, where blue-dominant pixels were removed to eliminate the blue mat used for improvement of contrast in top view images of small plants.

### 2.3. Affine image alignment using fourier-mellin phase correlation

Phase correlation between each two images is computed as Fourier inverse of the normalized cross-power spectrum (*CPS*):

(1) $$PC=F^{-1}(CPS),$$

where

(2) $$\begin{array}{l} \begin{array}{c} \alpha =F\left(A\right)\\ \beta =F\left(B\right) \end{array} \end{array}$$

are the complex Fourier transforms of the images *A* and *B* and

(3) $$\begin{array}{l} CPS=\frac{\alpha \beta^{*}}{\left|\alpha \beta^{*}\right|} \end{array}$$

is the so-called cross-power spectrum (CPS). According to the Fourier shift theorem, relative displacement (Δ*x*, Δ*y*) in the Cartesian system or coordinates (or, alternatively, scaling and rotation in the polar system of coordinates) between two otherwise structurally identical images, i.e.,

(4) $$\begin{array}{l} B_{x,y}=A_{x-\Delta x,y-\Delta y}, \end{array}$$

leads to phase-shift in the frequency domain

(5) $$\begin{array}{l} \beta_{u,v}={\text{e}}^{-2\pi i\varphi }\alpha_{u,v}, \end{array}$$

where $\varphi =\left(\frac{u\Delta x}{N}+\frac{v\Delta y}{M}\right)$ and is *N*×*M* are the image dimensions. As a consequence, the cross power spectrum between two identical images with a relative shift in the Cartesian system of coordinates (or scaled/rotated in the polar system of coordinates) describes the phase-shifts of the Fourier transform in the frequency domain:

(6) $$\begin{array}{l} CPS_{u,v}=\frac{\alpha_{u,v}{\text{e}}^{2\pi i\varphi }\alpha_{u,v}^{*}}{\left|\alpha_{u,v}{\text{e}}^{2\pi i\varphi }\alpha_{u,v}^{*}\right|}={\text{e}}^{2\pi i\varphi }. \end{array}$$

For two identical images with the relative spatial displacement (Δ*x*, Δ*y*), the inverse Fourier integral of (6) represents a *N*×*M* map exhibiting a single singularity at the point (*x* = Δ*x, y* = Δ*y*)

(7) $$\begin{array}{l} PC_{x,y}=\delta \left(x-\Delta x,y-\Delta y\right). \end{array}$$

**Figure 3:**
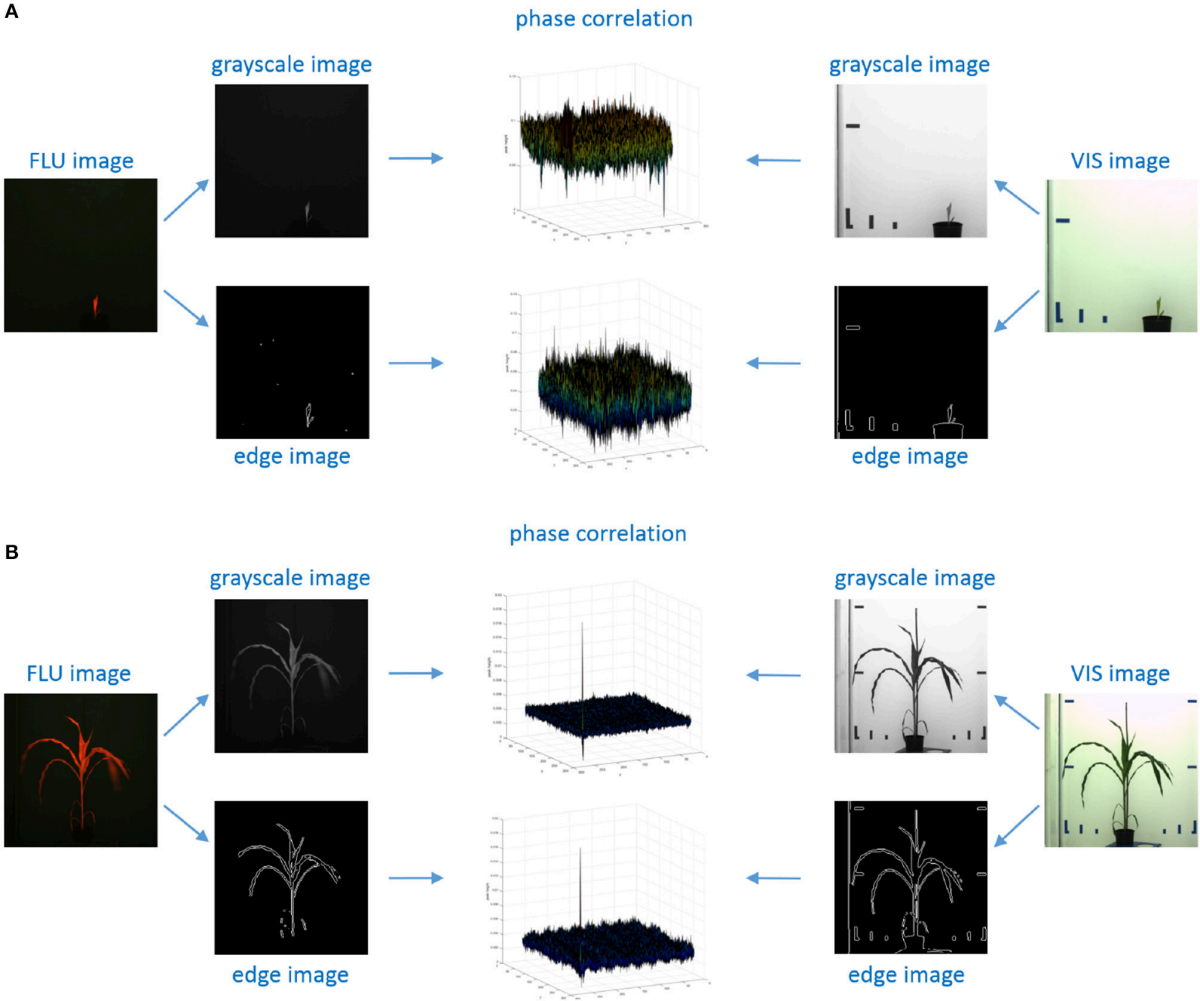
Examples of phase correlation between FLU and VIS images of young and small **(A)** vs. older/larger **(B)** maize shoots. For calculation of relative geometrical transformations, alternative PC registrations were performed using 2D grayscale and edge images that were derived from original FLU and VIS images, respectively. **(A)** Phase correlation between VIS/FLU images of small shoots exhibit a high level of noise with multiple maxima of nearly same height. **(B)** Correlation between large and unique patters in images of older plants lead to a single maximum peak corresponding to the relative image transformation.

This means that the maximum peak of phase correlation between two identical images yields the relative image translation in the Cartesian system of coordinates, or their relative scaling and rotation in polar coordinates, see examples in Figure 3.

Calculation of affine image transformations from Fourier-Mellin phase correlation was performed using a modified version of the MATLAB *imregcorr* routine which in addition to the affine transformation matrix returns the height of the maximum PC peak. For assessment of reliability of image transformation, a fixed threshold of *H* > 0.03 was used as suggested in Reddy and Chatterji (1996). Transformations obtained with *H* < 0.03 typically indicate a failure of PC registration, for example, due to low and missing structural similarities between two images.

### 2.4. Evaluation of image registration

To evaluate the results of image registration two criterions for characterization of algorithmic robustness and accuracy are introduced.

#### 2.4.1. Success rate of image registration

The success rate (SR) of image registration is calculated as the ratio between the number of successfully performed image registrations (*n*_*s*_) divided by the total number of registered image pairs (*n*):

(8) $$\begin{array}{l} SR=\frac{n_{s}}{n}. \end{array}$$

Thereby, the criterion of successful image alignment was defined by the minimum admissible height of the maximum PC peak (*H* > 0.03) as suggested by Reddy and Chatterji (1996) as well as reasonable bounds of image translation, rotation and scaling. Geometrical transformations that do not match these criterions were treated as failure of PC registration.

#### 2.4.2. Overlap ration of registered image regions

The second criterion is constructed to quantify the overlap ratio (OR) between the area of plant regions in VIS images that are covered by the registered FLU image (*a*_*r*_) and the total area of manually segmented plant regions (*a*):

(9) $$\begin{array}{l} OR=\frac{a_{r}}{a}. \end{array}$$

While SR serves as a criterion indicating that PC routine succeed in producing some reasonable transformation, OR describes the accuracy of successful transformations.

## 3. Results

### 3.1. Single-step PC registration of full-size images

First, PC registration of original, full-size FLU and VIS images of maize, wheat and arabidopsis shoots was performed using the conventional single-step PC approach. Thereby, eight different preprocessing variants including

**Figure 4:**
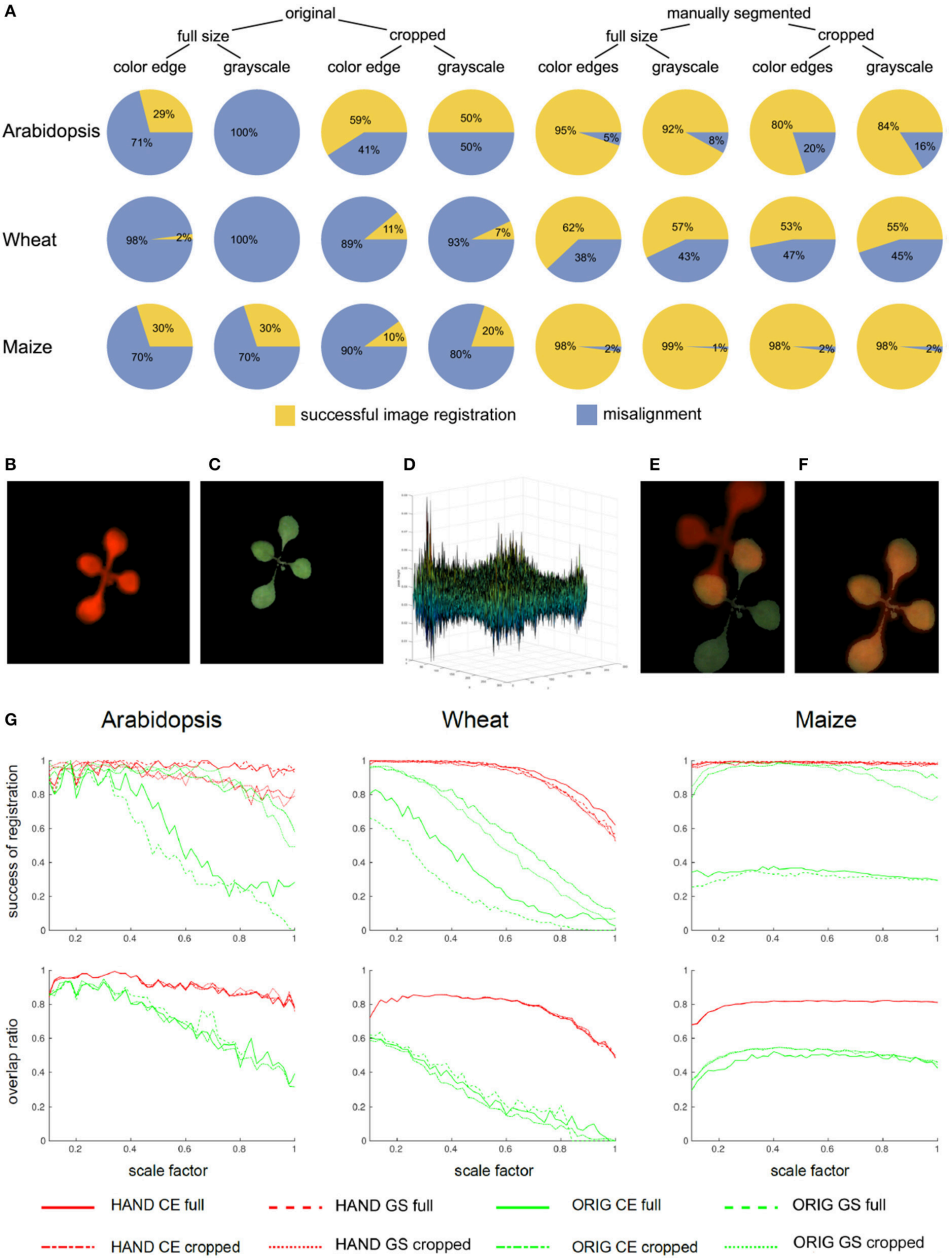
Statistics of single-step PC registration of original and preprocessed plant images. **(A)** Pie charts show differences in the relative success rate of PC registration (Equation 8) between different preprocessing conditions including full-size vs. cropped as well as original vs. manually segmented FLU and VIS images. Enhancement of image similarity by means of manual background elimination leads to substantial improvement of PC registration rate. However, cropping of target regions turned out to be not advantageous. Example of a pair of VIS/FLU images **(B,C)** demonstrates that PC of a young Arabidopsis shoot with very similar leaf exhibits multiple peaks **(D)**. As a consequence of noisy PC the maximum peak may not correspond to the optimal image alignment **(E)**. Downscaling effectively performs image smoothing which improves phase correlation. For the scale factor 0.32, an optimal image alignment was found **(F)**. **(G)** Plots of success rates and overlap ratios (Equation 9) of PC registration for original (ORIG), hand segmented (HAND full) and cropped (HAND cropped) grayscale (GS) and color-edge (CE) images as a function of scaling ratio [0.1, 1.0]. As one can see, downscaling has strong impact on accuracy of PC registration, however, the optimal scaling factor should not be too low and the optimum lays in the range between [0.3, 0.6]. The overlap ratio lower than 1, especially, for wheat and maize shoots means that single-step PC registration results in an alignment which does not produce a complete coverage of manually segmented plant region in the VIS image by the registered FLU mask.

- Gray-scale version of unprocessed full-size VIS/FLU images
- Color-edges version of unprocessed full-size VIS/FLU images
- Gray-scale version of unprocessed full-size and adaptively cropped VIS/FLU images
- Color-edges version of unprocessed full-size and adaptively cropped VIS/FLU images
- Gray-scale version of manually segmented full-size VIS/FLU images
- Color-edges version of manually segmented full-size VIS/FLU images
- Gray-scale version of manually segmented full-size and adaptively cropped VIS/FLU images
- Color-edges version of manually segmented full-size and adaptively cropped VIS/FLU images

were compared. To assess the performance of PC registration for different preprocessing conditions, cumulative statistics of successful image alignment was calculated for all days of each experiment. As one can see from Figure 4A, manual segmentation significantly improves the success rate of PC registration. Surprisingly, cropping of plant regions does not always improve and sometimes even worsens the PC performance. This rather unexpected result could be traced back to higher probability of misalignment of partially similar plant structures with the larger relative size in relationship to the size of (cropped) image. This was, in particular, observed in juvenile arabidopsis plants with only a few similar leaves. The relationship between the size of plant structures and the image size has, in turn, an impact on their spectral representation, i.e., different weights of lower and higher frequencies, which, in the case of partially similar, blurry and/or repetitive pattern can lead to maximization of PC peak related to locally optimal alignment. An example of such a case is shown in Figures 4B–F. We found that image downscaling can help to avoid such misalignments and to enhance the PC peak corresponding to globally optimal image registration, cf. Figure 4E vs. Figure 4F.

### 3.2. Effects of downscaling on robustness of PC image alignment

**Figure 5:**
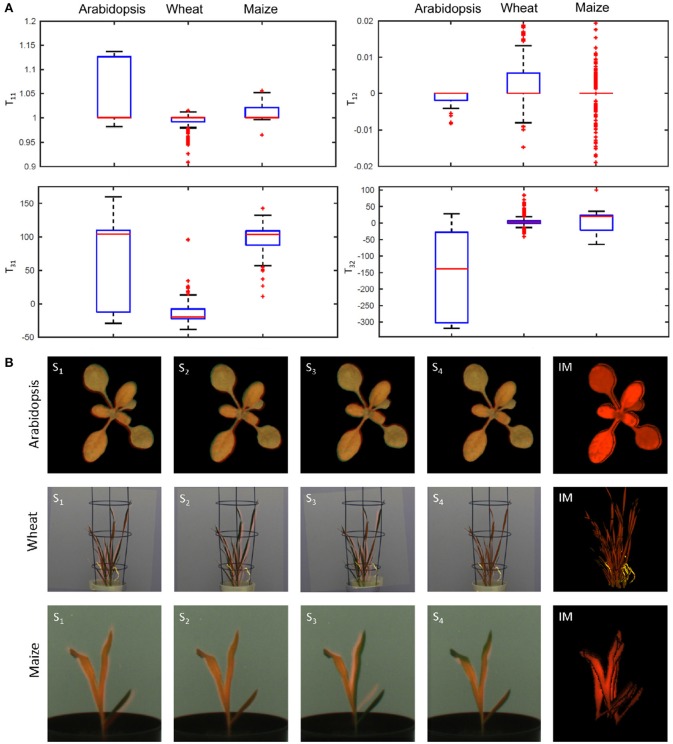
Ambiguity of affine image alignment due to non-uniform leaf motion. In addition to global geometrical transformations, such as image scaling, translations and rotations, plants exhibit uncorrelated motion of leaves which can not be compensated by a single affine alignment. **(A)** Diagonal (*T*_11_), off-diagonal (*T*_12_), and translational components (*T*_31_, *T*_32_) of the affine transformation matrix determined for different scale factors undergo considerable variation which reflects ambiguity of affine alignment of structurally non-identical images. **(B)** Single-step PC registrations of differently scaled images (S_*i*_) may lead to partial overlaps of different parts of the plant. In order to improve accuracy of PC registration of non-uniformly moving plant structures, the results of multiple registrations are integrated into a single integrated mask (IM) which provides significantly better coverage of the entire plant shoot in FLU and VIS images.

In order to systematically analyzed the effects of image downscaling on robustness of PC registration, tests with downscaled images in the range of scaling factors between [0.1, 1.0] and the step-size 0.02 were performed. Plots in Figure 4G show the success rate (Equation 8) and the overlap ratio (Equation 9) as a function of scale factor. As one can see, downscaling improves both accuracy and robustness of PC registration. However, the robust algorithmic performance is achieved in the range of intermediate scaling factors [0.3, 0.6] that probably correspond to the optimal degree of image smoothing. Detailed analysis of geometrical transformations calculated for differently scaled images reveals that they correspond to optimal registration of some but not all leaves. Consequently, all components of the affine transformation matrix that stand for the relative image scaling, translation, and rotation undergo variations, see Figure 5A. This sort of locally-optimal alignment is particularly evident for plants exhibiting a non-uniform motion, for example, due to uncorrelated leaf movements that occur, for example, shortly after abrupt stop of carriers, e.g., after movements or rotations.

### 3.3. Integration of multiple PC registrations into a single mask

Since downscaling of images with different scaling factors results in slightly different geometrical transformations that tend to be locally- but not globally-optimal, integration of a series of PC registrations into a single registration mask was introduced. Figure 5B shows examples of single-step locally-optimal image alignments followed by their integration into a single integrated mask (see the right raw). Using the iterative PC strategy, the overlap ratio of 100% between integrated FLU mask and VIS regions was achieved for all images of three different experiments with arabidopsis, wheat, and maize shoots.

### 3.4. Dependency of PC performance on plant growth

**Figure 6:**
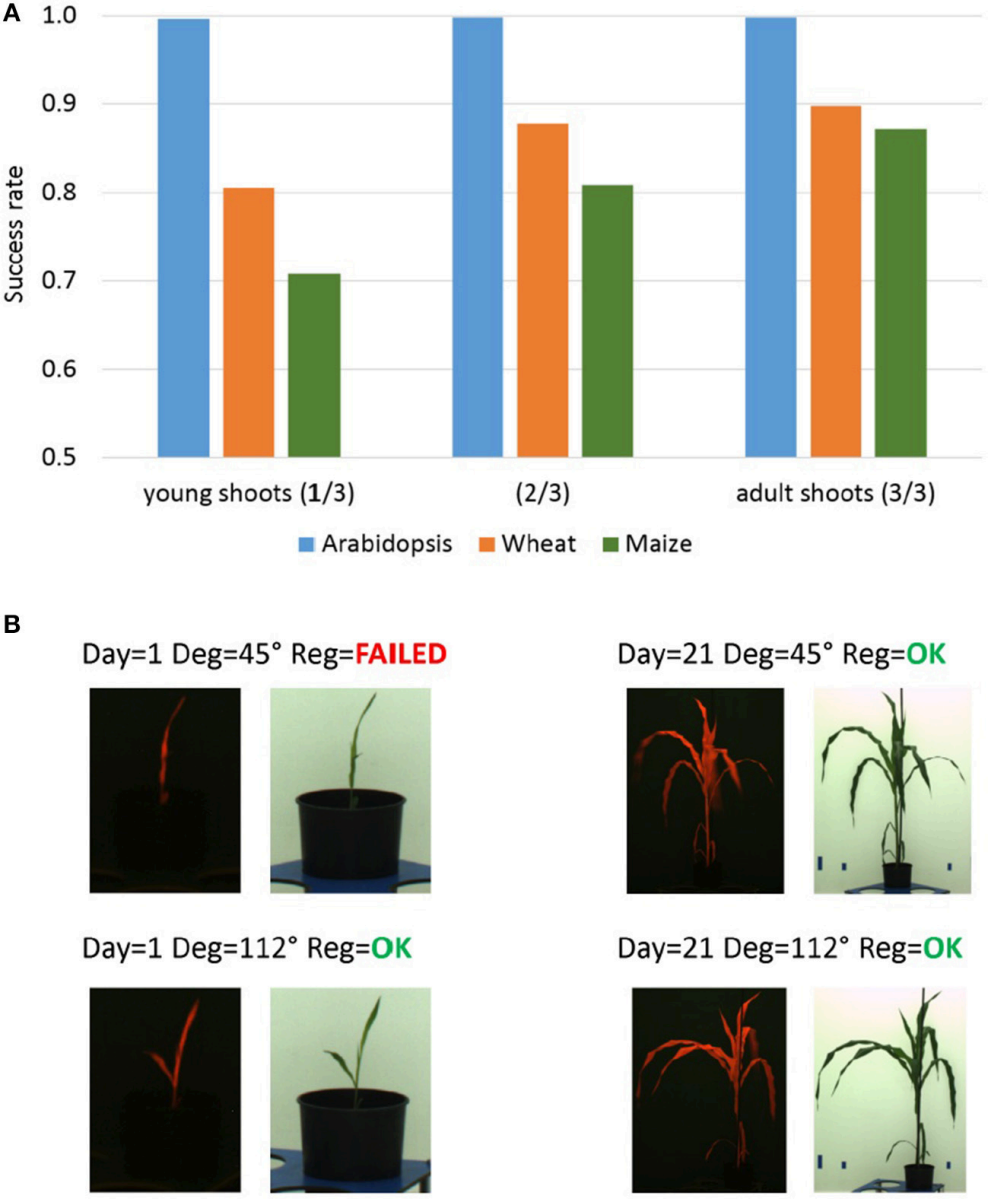
Dependency of the PC registration on plant growth. **(A)** Median success rates of single-step PC registration of FLU/VIS images for three plant age/growth phases of Arabidopsis (top-view), wheat and maize (side-view) phenotyping experiments including young (1. phase), intermediate stage (2. phase) and adult shoots (3. phase). Reduced success rate of PC registration is caused by a more frequent occurrence of non-specific shape of young wheat and maize shoots in side-view from certain rotation angles. **(B)** Examples of FLU/VIS images of young and adult maize shoots taken from different rotation angles. The redundant shape (“thin vertical line”) of the young maize shoot in the FLU/VIS image pair taken from the rotation degree 45° does not exhibit unique spectral characteristics causing a failure of the PC registration.

As the accuracy of PC registration is essentially dependent on unique spectral characteristics of target plant structures, a reduced PC performance was observed for young plant shoots exhibiting redundant shapes (e.g., thin vertical lines, blobs, etc.). Similar to the problem of multiple similar leaves, non-specific shape of plant shoots causes ambiguity and inaccuracy of the PC image alignment. Figure 6A summarizes success rates of the PC registration calculated for three age/growth phases of arabidopsis, wheat and maize phenotyping experiments including young, intermediate stage and adult plant shoots. As one can see, success rate of the PC registration of wheat and maize shoots gradually improves with the plant age (i.e., phase of experiment). Figure 6B gives examples of successful and failed image registration of young and adult maize shoots. From certain views (here, for example, the rotation degree 45°), young maize shoots exhibit a non-specific shape (“thin vertical line”) similar to some non-plant structures (e.g., boundaries of carriers, background markers, etc.). Obviously, it is the combination of several factors (i.e., optical plant appearance (shape/size) at certain developmental stages from certain views, and the presence of non-plant background structures) which causes dependency of the PC performance on plant age/growth in our setup.

## 4. Conclusion

Here, we approached the problem of multi-modal plant image registration using the Fourier-Mellin phase correlation technique. We began this explorative study with assumption that FLU/VIS image registration can be performed using a global affine image transformation. Our investigations showed, however, that structural differences and non-uniform image motion between FLU/VIS plant images require substantial extensions of the conventional single-step PC approach. Our experimental tests with large amount of different plant images confirm previous observations that PC registration of multi-modal non-identical images is sensitive to structural noise and ambiguous image content which can be caused by repetitive self-similar plant structures, combination of young shoots with non-specific shape and background structures, image blurring or non-uniform motion due to frequently observed inertial leaf movements. Some of these problems can be avoided by optimization of the optical scene and the measurement protocol. For example, homogenization and elimination of complexity of background regions as well as longer relaxation times after relocation of plants from VIS to FLU chambers will certainly be helpful. We demonstrate that the accuracy of PC registration can be improved when PC is applied to appropriately preprocessed and downscaled images that exhibit higher degree of structural similarity such as color-edge and background-filtered images. In contrast, cropping of target regions may be counterproductive as it enhances spectral differences between non-identical images and makes phase correlation rather noisy. Strictly speaking, non-uniform image motion represents a non-rigid image transformation which goes beyond the scope of applicability of the affine PC-based registration. To overcome this limitation, we introduced an extension to the conventional single-step PC which is based on integration of a series of locally-optimal PC registrations resulting from alignment of differently scaled images. Suggested iterative scheme for calculation of an integrated registration mask turned out to provide a significantly better overlap between registered FLU and VIS images in the case of non-uniform leaf motion. The disadvantage of the present algorithmic implementation consists in computationally inefficient search for different locally-optimal image transformations in the scale space. Alternative algorithmic approaches are required for a more efficient detection of the relevant peaks of a noisy phase correlation. In summary, our extended PC scheme represents a promising approach to fully automated alignment and segmentation of optically complex and heterogeneous multi-modal plant images suitable for application within the scope of high-throughput plant image analysis and phenotyping.

## Author contributions

MH and EG conceived, designed and performed the computational experiments, analyzed the data, wrote the paper, prepared figures and tables, and reviewed drafts of the paper. AJ and KN executed the laboratory experiments, acquired image data, co-wrote the paper, and reviewed drafts of the paper. TA co-conceptualized the project, and reviewed drafts of the paper.

### Conflict of interest statement

The authors declare that the research was conducted in the absence of any commercial or financial relationships that could be construed as a potential conflict of interest.
